# CXCL10 is a crucial chemoattractant for efficient intranasal delivery of mesenchymal stem cells to the neonatal hypoxic-ischemic brain

**DOI:** 10.1186/s13287-024-03747-8

**Published:** 2024-05-07

**Authors:** Eva C. Hermans, Vanessa Donega, Cobi J. Heijnen, Caroline G.M. de Theije, Cora H. Nijboer

**Affiliations:** 1 Department for Developmental Origins of Disease, University Medical Center Utrecht Brain Center and Wilhelmina Children’s Hospital, Utrecht University, Internal post: KC03.068.0, PO Box 85090, Utrecht, 3508 AB The Netherlands; 2 Anatomy & Neurosciences, Amsterdam UMC, location Vrije Universiteit Amsterdam, De Boelelaan 1117, Amsterdam, The Netherlands; 3 Amsterdam Neuroscience, Cellular and Molecular Mechanisms, Amsterdam, The Netherlands; 4 Department of Psychological Sciences, Rice University, Houston, TX USA

**Keywords:** Mesenchymal stem cell therapy, Neonatal Hypoxic-Ischemic Encephalopathy, Migration, CXCL10, Intranasal

## Abstract

**Background:**

Hypoxic-Ischemic Encephalopathy (HIE) is a leading cause of mortality and morbidity in newborns. Recent research has shown promise in using intranasal mesenchymal stem cell (MSC) therapy if administered within 10 days after Hypoxia-Ischemia (HI) in neonatal mice. MSCs migrate from the nasal cavity to the cerebral lesion in response to chemotactic cues. Which exact chemokines are crucial for MSC guidance to the HI lesion is currently not fully understood. This study investigates the role of CXCL10 in MSC migration towards the HI-injured brain.

**Methods:**

HI was induced in male and female 9-day-old C57BL/6 mice followed by intranasal MSC treatment at day 10 or 17 post-HI. CXCL10 protein levels, PKH26-labeled MSCs and lesion size were assessed by ELISA, immunofluorescent imaging and MAP2 staining respectively. At day 17 post-HI, when CXCL10 levels were reduced, intracranial CXCL10 injection and intranasal PKH26-labeled MSC administration were combined to assess CXCL10-guided MSC migration. MSC treatment efficacy was evaluated after 18 days, measuring lesion size, motor outcome (cylinder rearing task), glial scarring (GFAP staining) and neuronal density (NeuN staining) around the lesion. Expression of the receptor for CXCL10, i.e. CXCR3, on MSCs was confirmed by qPCR and Western Blot. Moreover, CXCL10-guided MSC migration was assessed through an in vitro transwell migration assay.

**Results:**

Intranasal MSC treatment at day 17 post-HI did not reduce lesion size in contrast to earlier treatment timepoints. Cerebral CXCL10 levels were significantly decreased at 17 days versus 10 days post-HI and correlated with reduced MSC migration towards the brain. In vitro experiments demonstrated that CXCR3 receptor inhibition prevented CXCL10-guided migration of MSCs. Intracranial CXCL10 injection at day 17 post-HI significantly increased the number of MSCs reaching the lesion which was accompanied by repair of the HI lesion as measured by reduced lesion size and glial scarring, and an increased number of neurons around the lesion.

**Conclusions:**

This study underscores the crucial role of the chemoattractant CXCL10 in guiding MSCs to the HI lesion after intranasal administration. Strategies to enhance CXCR3-mediated migration of MSCs may improve the efficacy of MSC therapy or extend its regenerative therapeutic window.

**Supplementary Information:**

The online version contains supplementary material available at 10.1186/s13287-024-03747-8.

## Graphical Abstract

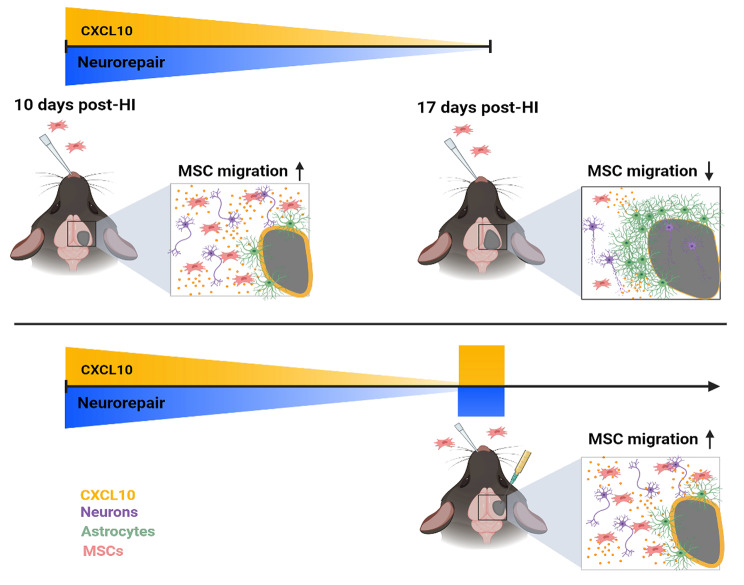

## Background

Perinatal asphyxia is a major cause of Hypoxic-Ischemic Encephalopathy (HIE) in the newborn. HIE is a significant contributor to neonatal mortality and can result in lifelong morbidities including mental retardation, seizures and cerebral palsy [1]. In developed countries the incidence of HIE is estimated as 1–2 per 1000 live births [2]. HIE results from a cascade of damaging events in the brain leading to neuronal death over hours to days after the insult. These events include oxidative stress, excitotoxicity, apoptotic cell death and microglial activation [1]. Interestingly, the damaging processes evoked by Hypoxia-Ischemia (HI) can continue for days to weeks after the insult [1]. Cytokines and chemokines play crucial roles in this process, regulating increased inflammatory cell traffic and promoting cell migration [1, 3].

Currently, hypothermia is the only clinically applied therapy shown to be modestly effective in the treatment of HIE. However, hypothermia can only be applied in cases of moderate to severe brain injury and most importantly, hypothermia only has a short treatment window of 6 h [4, 5]. Novel efficacious therapies specifically aimed at repair of the brain at later stages after the HI insult are urgently needed.

In the past decade, mesenchymal stem cell (MSC) therapy has emerged as a promising treatment strategy for HIE. MSCs can be collected from multiple sources, such as bone marrow (BM), umbilical cord blood, umbilical cord tissue (Wharton’s jelly) or adipose tissue [6, 7]. MSCs in BM were identified first, are well-characterized and are the most commonly used source of MSCs, also in clinical trials [6, 7]. In this study we used murine BM-MSCs as this is the most often used MSC source applied intranasally in experimental HI animal models including in our own previous work [6, 8–11]. Previous research showed that upon non-invasive intranasal administration, BM-MSCs can migrate towards the HI-induced lesion in the Vannucci-Rice mouse model of neonatal HI brain injury [8–10, 12–15]. Using the same experimental model, intranasal BM-MSC treatment has been shown to reduce the cerebral lesion size and to improve motor and cognitive functioning [8, 10, 11, 16]. Notably, intranasal BM-MSC treatment appears to have a long therapeutic window, being effective at least until 10 days after the insult [10]. Further postponing intranasal BM-MSC therapy abolishes the treatment efficacy [10, 17] indicating a limit to the therapeutic window. Interestingly, our previous study showed that at 17 days post-HI cerebral mRNA expression of a number of chemokines, among which chemokine-X-C motif chemokine Ligand 10 (*cxcl10)*, was strongly downregulated compared to 10 days after the insult [9]. Therefore, CXCL10 may play a role in the efficacy of intranasal MSC treatment possibly by facilitating MSC migration.

CXCL10 is a small protein (8–15 kilodalton (kDa)) that binds to C-X-C Motif Chemokine Receptor 3 (CXCR3), a 7-transmembrane G protein-coupled receptor [18]. CXCR3 activation by CXCL10 activates various downstream pathways including Proto-oncogene tyrosine-protein kinase Src (Src), Phosphoinositide 3-kinase (PI3K), Extracellular signal-regulated protein kinase 1 and 2 (Erk1/2), and Mitogen activated protein kinase (MAPK) signalling [19]. While CXCR3 also serves as a receptor for C-X-C motif chemokine Ligand 9 (CXCL9) and C-X-C motif chemokine Ligand 11 (CXCL11) that structurally resemble CXCL10, CXCR3 is the only known receptor for CXCL10 [18, 19]. *Cxcl10* RNA and CXCL10 protein levels are upregulated after neonatal brain injury in rats and piglets [20, 21] suggesting a potential role of CXCL10 in neonatal brain injury. Upon brain injury, CXCL10 is primarily secreted by astrocytes, microglia and neurons and attracts peripheral or meningeal immune cells to the lesion [22–24]. Similarly, MSCs have the capability to migrate towards CXCL10 and may be attracted in a comparable way as immune cells to the injured brain [25, 26]. In the current study we examined the role of CXCL10 as a potential chemoattractant for BM-MSC migration towards the HI-injured brain. To elucidate the role of the CXCL10-CXCR3 pathway in MSC migration, we first assessed CXCR3 expression on MSCs and CXCL10-mediated MSC migration in vitro. Additionally, we investigated in a proof-of-principle study in neonatal HIE mice whether intracranial CXCL10 injection drives effective MSC delivery and repair of the HI lesion at a timepoint beyond the therapeutic window of 10 days post-HI.

## Materials and methods

### HI induction in neonatal mice

The experiments are reported in compliance with the Animal Research: Reporting of In Vivo Experiments (ARRIVE) guidelines [27].

C57Bl/6 mice (OlaHsa, ENVIGO, Horst, The Netherlands) were kept in standard housing conditions with woodchip bedding, cardboard shelters and tissues provided on a 12 h day/night cycle (lights on at 7:00 am) in a temperature-controlled room at 20–24 °C and 45–65% humidity with ad libitum food and water access. Two days after induction of HI injury, litters received cage enrichment: a turning wheel on a plastic shelter. Mice were bred in-house by placing wild type males and females together in a ratio of 1:1 or 1:2 for 4 days. Afterwards, dams were housed solitarily to give birth. Day of birth was considered as postnatal day (P) 0.

Neonatal HI injury was induced at P9 by unilateral carotid artery ligation under isoflurane anesthesia ((5–10 min; 5% induction, 3–4% maintenance with flow O_2_: air 1:1) followed by recovery with their mother for at least 75 min. Xylocaine (#N01BB02, AstraZeneca, Cambridge, UK) and Bupivacaine (#N01BB01, Actavis, Allergan Inc, Dublin, Ireland) were applied to the wound for pre- and post-operative analgesia. After recovery, pups were subjected to systemic hypoxia at 10% O_2_ for 45 min in a temperature-controlled and humidified hypoxic incubator. Control sham-operated (SHAM) littermates were subjected to anesthesia and surgical incision only. Exact numbers of animals used in each experiment are depicted in the figure legends. In total 141 animals were used in this study. All experimental groups were randomly divided per litter. Three animals were excluded from the study because they reached the humane endpoint a priori defined as < 3.5 g weight gain from P9 to P26 (1 HI intracranial (i.c.) PBS + intranasal (i.n.) PBS animal, 2 HI i.c. CXCL10 + i.n. MSC animals). Group sizes were determined by performing power analysis based on treatment effect size obtained from previous experiments. Primary outcome measure of this study was MAP2 tissue loss.

### Intracranial CXCL10 injections

At 17 days post-HI surgery, HI mice were anesthetized (isoflurane 5–10 min; 5% induction and 3–4% maintenance with flow O_2_: air 1:1), skin of the head was opened up and a hole was made in the skull manually with a 20 gauge needle, followed by an intracranial injection with 1 μg/2μL CXCL10 protein (300 − 12, Peprotech, London, UK) or 2μL vehicle (D-PBS, Merck KGaA, Darmstadt, Germany) into the ipsilateral hemisphere at 2 mm caudal to bregma, 2 mm right from midline and 2 mm below dural surface (Supplementary Fig. 1) using a stereotactic frame (Stoelting, Illinois, USA). Afterwards, skin was sutured and Xylocaine and Bupivacaine were applied subcutaneously for pre- and post-operative analgesia. Carprofelican (Dechra, ‘s-Hertogenbosch, The Netherlands) was administered as systemic analgesia subcutaneously immediately after and on the two consecutive days after the surgery.

### MSC culture and PKH26-labeling

Murine (C57BL/6) BM mesenchymal stem cells (GIBCO, S1502-100, Thermo Fisher Scientific, Bleiswijk, The Netherlands) were used for all experiments. Cells were cultured in MSC medium containing DMEM: F12 GlutaMax (31331093, Fisher Scientific, Landsmeer, The Netherlands), 10% Fetal Calf Serum (FCS, 10270106, Fisher Scientific), 0.05% gentamycin (15710064, Fisher Scientific) and 1% penicillin/streptomycin (P/S, 15140163, Fisher Scientific) in T75 flasks (353110, Corning Life Sciences, Amsterdam, The Netherlands). MSCs were passaged once before in vitro and in vivo use, according to the manufacturer’s instructions. Cells were cultured at 37 °C, 5% CO_2_ and 90% humidity. For PKH26^+^ labelling, MSCs were labelled with the PKH26 Red fluorescent cell linker kit according to manufacturer’s protocol prior to administration (PKH26GL, Merck KGaA).

### (Intranasal) use of MSCs

**Fig. 1:**
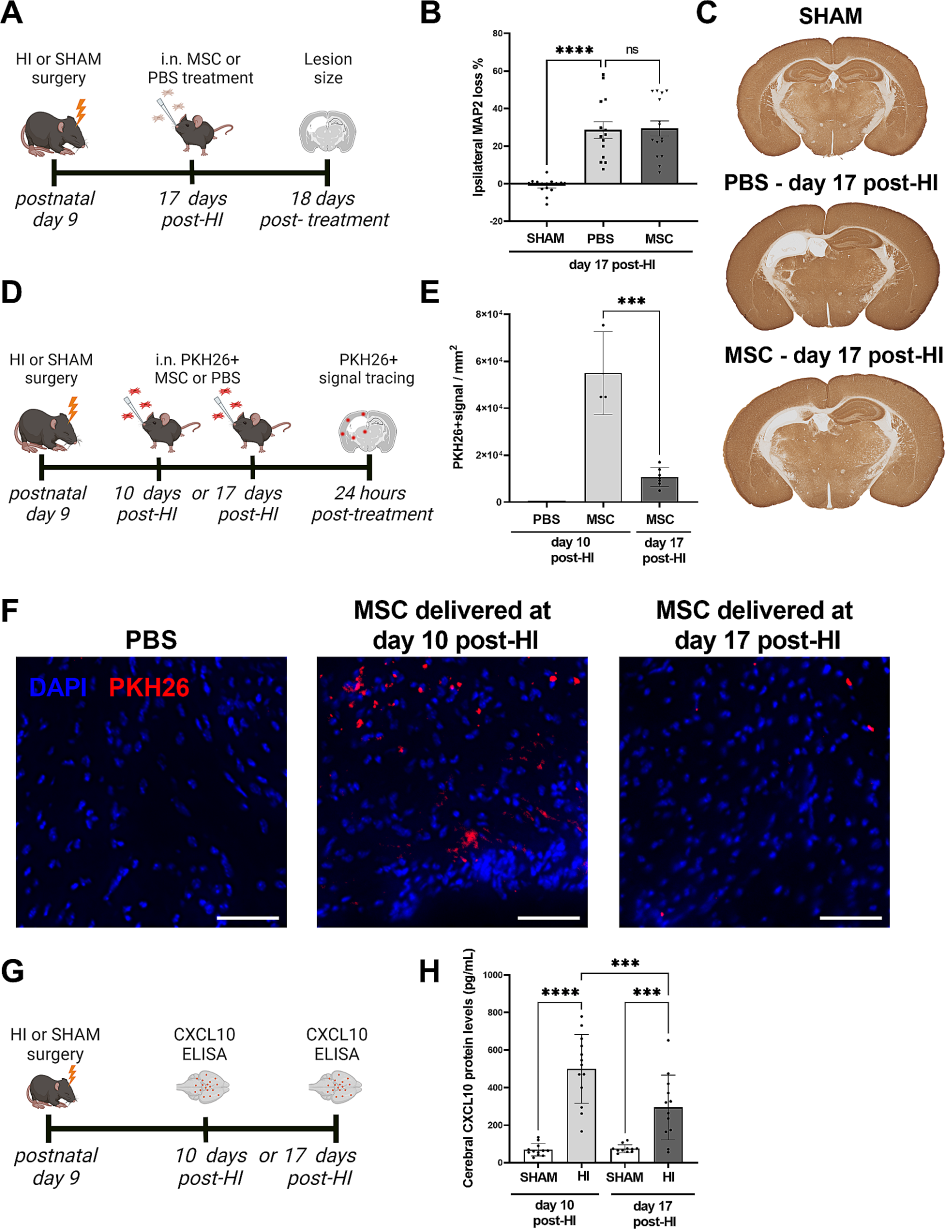
Non-efficacious MSC treatment 17 days post-HI coincides with reduced MSC migration and cerebral CXCL10 levels. **A**) Experimental set-up of B-C. **B**) Quantification of ipsilateral MAP2 loss (%), as a measure of lesion size, in SHAM and HI littermates treated with PBS or MSCs at day 17 post-HI; SHAM: *n* = 14 (7♀, 7♂), PBS: *n* = 14 (7♀, 7♂), MSC: *n* = 15 (6♀, 9♂). (**C**) Representative examples of MAP2 staining in brains of SHAM-controls and HI animals treated intranasally with PBS or MSCs at 17 days post-HI. Lack of MAP2 staining (white) shows the lesion. (**D**) Experimental set-up of E-F. (**E**) Quantification of average PKH26^+^ signal in pixels per mm^2^, as an estimation of MSC migration, in HI animals treated with PBS (negative control) or PKH26-labeled MSCs at day 10 or day 17 post-HI; PBS: *n* = 1(1♂), MSCs day 10: *n* = 3(1♀,2♂), MSCs day 17: *n* = 7(3♀, 4♂). (**F**) Representative example of perilesional area (cortex) showing PKH26 staining (red) at 24 h after i.n. PKH26-labeled MSC delivery at day 10 or 17 post-HI (20x magnification). Counterstain with DAPI (blue). Scale: 50 μm. (**G**) Experimental set-up of H. (**H**) CXCL10 protein levels (pg/mL) in ipsilateral brain homogenates of HI and SHAM-control animals at day 10 and 17 post-HI; SHAM-day 10: *n* = 12(5♀, 7♂), HI-day 10: *n* = 13(5♀, 8♂), SHAM-day 17: *n* = 11(4♀, 7♂), HI-day 17: *n* = 12(5♀, 7♂). *** *p* < 0.001 **** *p* < 0.0001. ns, non-significant. Data is presented as mean ± SEM. i.n. = intranasal

**Fig. 2:**
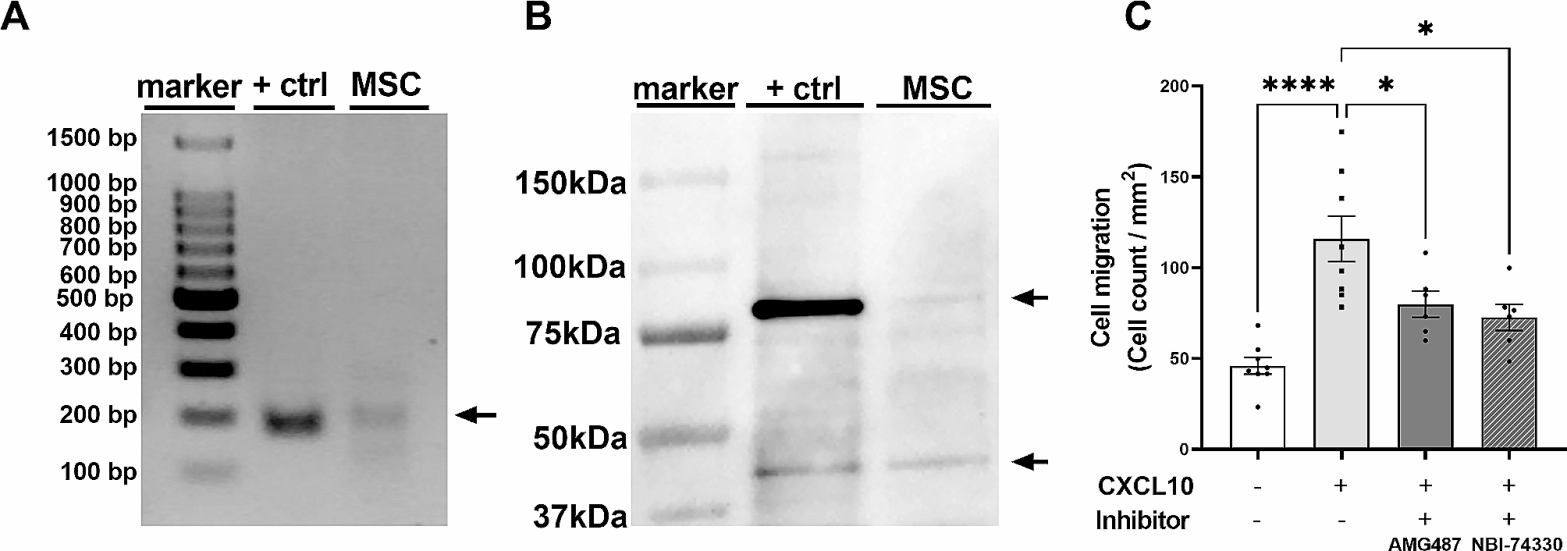
Murine BM-MSCs express CXCR3 and can migrate towards CXCL10 via CXCR3. (**A**) qPCR product of CXCR3 transcript in BV2 cells (positive ctrl) and MSCs. Arrow indicates representative qPCR product of 170 bp. Full-length gel is presented in Supplementary Fig. 2. (**B**) Western blot showing CXCR3 protein expression in the plasma membrane of MSCs. Protein bands corresponding to CXCR3 at 40 kDa and 80 kDa in BV2 cells (positive ctrl) and MSCs, indicated by the arrows. Full-length blot is presented in Supplementary Fig. 2. (**C**) Quantification of MSC migration in a transwell migration assay. Number of MSCs (cell count/per mm^2^) that migrated towards the stimulus (CXCL10), with or without CXCR3 inhibitors 1μM AMG487 or 100 nM NBI-74330. -CXCL10/+CXCL10: *n* = 8 wells, +CXCL10 + inhibitors: *n* = 6 wells. * *p* < 0.05, **** *p* < 0.0001. bp: base pairs. Data is presented as mean ± SEM. Ctrl = control

**Fig. 3:**
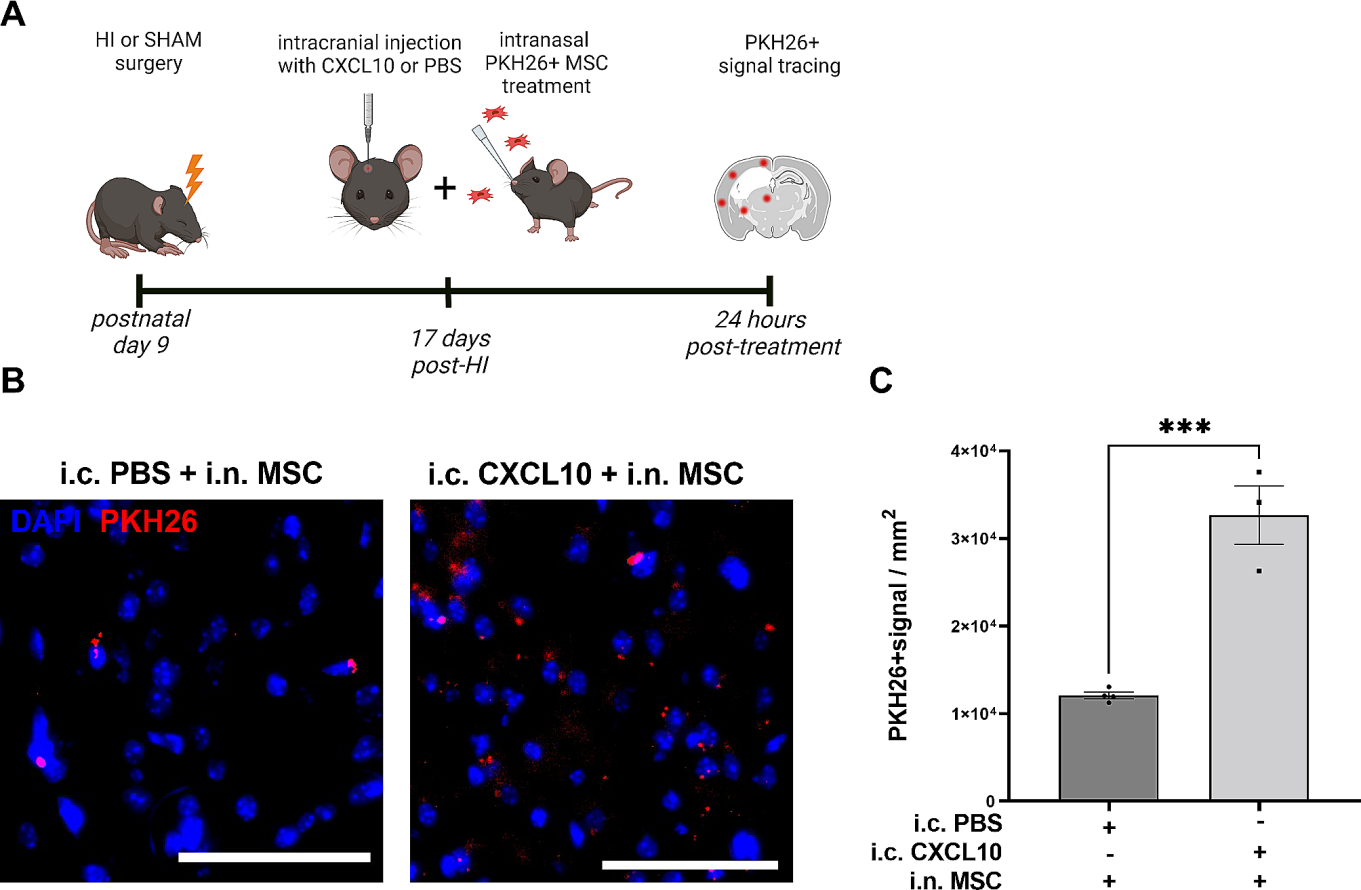
Increasing cerebral CXCL10 levels rescues MSC migration to the HI lesion at 17 days post-HI. (**A**) Experimental set-up. (**B**) Example pictures of PKH26^+^ signal (red) in the perilesional area (cortex) indicating PKH26^+^ MSC migration at 24 h after i.n. delivery at 17 days post-HI when combined with i.c. CXCL10 or PBS injection. Cell nuclei are counterstained with DAPI (blue), magnification 20x, Scale bar: 100 μm. (**C**) Quantification of PKH26^+^ signal in pixels per mm^2^ in the perilesional area after PKH26-labeled MSCs were delivered intranasally in combination with i.c. PBS (*n* = 4;1♀, 3♂) or CXCL10 (*n* = 3; 3♂). *** *p* < 0.001. Data is presented as mean ± SEM. i.c. = intracranial

**Fig. 4:**
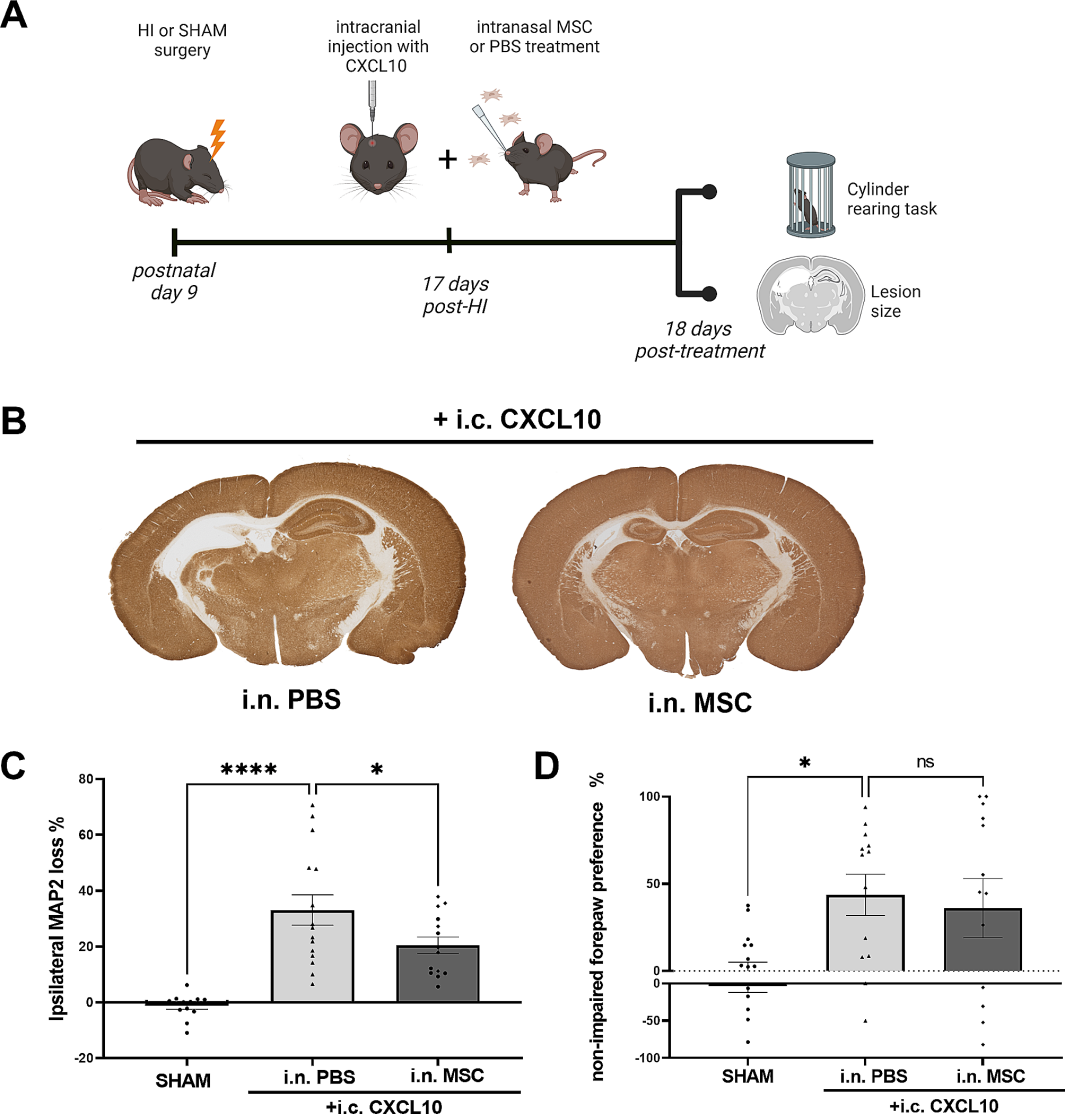
CXCL10 enhances efficacy of delayed MSC therapy on lesion size but not on functional outcome. (**A**) Experimental set-up. (**B**) Representative examples of MAP2 staining in brains of HI animals treated i.n. with PBS or MSCs at 17 days post-HI after i.c. CXCL10 injection. Lack of MAP2 staining (white) shows the lesion. (**C**) Quantification of ipsilateral MAP2 loss, as an indication of lesion size in SHAM animals (*n* = 14;7♀, 7♂), HI animals treated with i.c. CXCL10 + i.n. PBS (*n* = 15; 5♀, 10♂) and HI animals treated with i.c. CXCL10 + i.n. MSCs (*n* = 14; 6♀, 8♂). (**D**) Non-impaired forepaw preference at 18 days after treatment (35 days post-HI) in SHAM animals (*n* = 14;7♀, 7♂) and HI animals treated with i.c. CXCL10 + i.n. PBS (*n* = 13; 5♀, 8♂) or i.c. CXCL10 + i.n. MSCs (*n* = 13; 6♀, 7♂) at 17 days post-HI. *p < 0.05; ****p < 0.0001, ns: non-significant; i.c. = intracranial; i.n. = intranasal. Data is presented as mean ± SEM

**Fig. 5:**
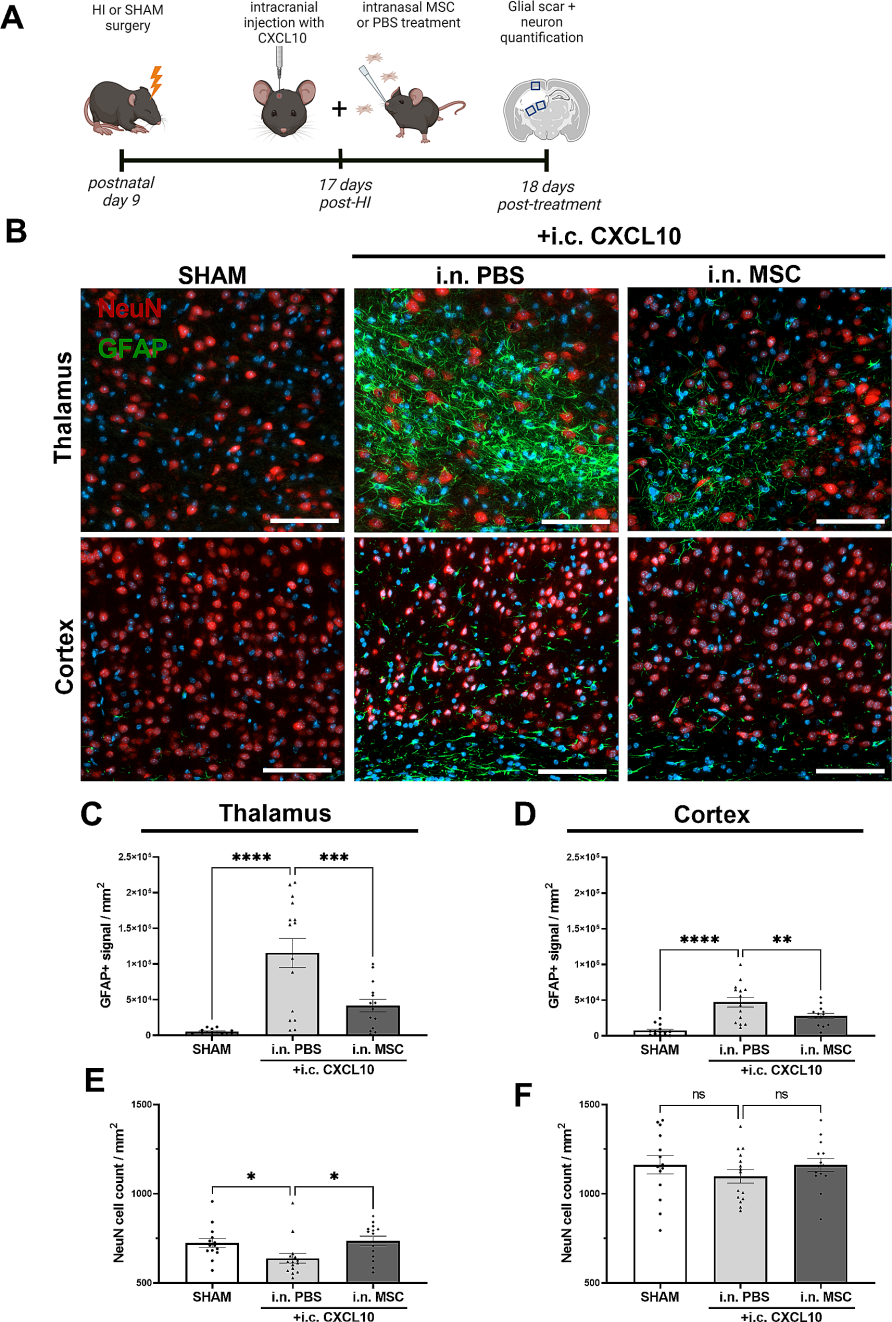
Reduced perilesional glial scarring and increased neuronal numbers after late intranasal MSC treatment in CXCL10-enriched brain. (**A**) Experimental set-up. (**B**) Representative pictures of NeuN^+^ neurons (red) and GFAP^+^ astrocytes (green) in regions ventral (thalamic region) and dorsal (cortical region) of the lesion in animals treated with i.c CXCL10 + i.n. PBS or i.n. MSCs. Magnification 10x, scale: 100 μm. DAPI (blue) was used as a nuclear counterstain. (**C**) Quantification of perilesional GFAP^+^ signal per mm^2^ ventral to the lesion (thalamic region). (**D**) Quantification of perilesional GFAP^+^ signal per mm^2^ dorsal to the lesion (cortical region). (**E**) Quantification of perilesional NeuN^+^ cell count per mm^2^ ventral to the lesion (thalamic region). (**F**) Quantification of perilesional NeuN^+^ cell count per mm^2^ dorsal to the lesion (cortical region). SHAM: *n* = 14 (7♀, 7♂), HI i.c. CXCL10 + PBS: *n* = 15 (5♀, 10♂), HI i.c. CXCL10 + i.n. MSCs: *n* = 14 (6♀, 8♂). * *p* < 0.05,**p < 0.01, ***p < 0.001, ****p < 0.0001. ns = non-significant. Data is presented as mean ± SEM. i.n. = intranasal; i.c. = intracranial

For PKH-based MSC tracing experiments at day 10 versus day 17 post-HI (Fig. 1), animals were intranasally treated with 1 × 10^6^ PKH26-labeled MSCs or vehicle (D-PBS). For the in vitro experiments of Fig. 2 non-labeled MSCs were used. For the CXCL10-guided MSC tracing experiments at day 17 post-HI, either 1 × 10^6^ PKH26-labeled (Fig. 3) or 0.5 × 10^6^ unlabeled MSCs (Figs. 4 and 5) were administered intranasally after a maximum of 6 hours after intracranial (i.c.) CXCL10 or vehicle injection. In all in vivo experiments, 30 min before intranasal MSC administration, hyaluronidase (100U, H4272, Merck KGaA) was administered intranasally to increase permeability of the connective tissue in the nasal cavity. MSCs were suspended in D-PBS and treatment was given by administration of 2 rounds of 2 droplets of 3 μL per nostril (12 μL in total).

### Sensorimotor outcome

Unilateral sensorimotor impairment was measured in the cylinder rearing test (CRT) [28] at 18 days after treatment (i.e. 35 days post-HI). Mice were placed in a transparent plexiglas cylinder and video-recorded for 5 min. Mice were omitted from the study when they failed to perform at least 10 weightbearing full rearings in 5 min (omissions: HI i.c. CXCL10 + i.n. VEH *n* = 2; HI i.c. CXCL10 + i.n. MSC: *n* = 1). This exclusion criterium was made *a priori.* The first weight-bearing paw contacting the cylinder wall during a full rear was scored by an experienced observer in a blinded manner as left (impaired), right (unimpaired) or both, using Noldus Observer XT16.0 software (Noldus, Wageningen, The Netherlands). Non-impaired paw preference was calculated as ((right rearings – left rearings)/ (right + left + both rearings)) x 100%.

### Analysis of MSC tracing

For PKH-based MSC tracing experiments, animals were terminated at 24 h after intranasal MSC delivery by an overdose of pentobarbital (Alfasan, Woerden, The Netherlands). Animals were transcardially perfused with PBS (Phosphate-buffered-saline, 524650-1, VWR, Radnor, Pennsylvania, USA) followed by 4% PFA (VWRK4078-9020, VWR). Brains were post-fixed in 4% PFA for at least 24 h and thereafter cryoprotected in an increasing sucrose gradient (10 to 30%) and frozen after embedment in an OCT compound (VWR). Coronal cryosections of mouse brains (8 μm) were cut at hippocampal level (comparable to bregma − 1.25 mm for adult mouse brain) and counterstained with 4′,6-diamidino-2-phenylindole (DAPI, D9542, Merck KGaA). Two different microscope set-ups were used for the different PKH26-labeled MSC tracing experiments due to unavailability of the first system at the time of the second experiment. Fluorescent images in Fig. 1 were captured at 20X magnification with an Olympus objective using a EMCCD camera (Leica Microsystems, Amsterdam, The Netherlands) and Softworx software (Applied Precision, Washington, USA). Fluorescent images in Fig. 3 were captured at 20x magnification with an Axio Observer Microscope equipped with Zen software equipped with AxioCam MRm (Carl Zeiss, Weesp, The Netherlands). Fluorescent Images were taken surrounding the lesion at all sites where PKH signal was present. The area of all positive pixels per mm^2^ was quantified by a blinded observer using FIJI 1.53 (National Institutes of Health, NIH, Bethesda, Maryland, USA).

### Histology

For therapeutic efficacy experiments (Figs. 1 and 4), animals treated with non-labeled MSCs were terminated 18 days after intranasal (i.n.) MSC administration by overdose of pentobarbital followed by transcardial perfusion with PBS and 4% PFA. Brains were post-fixed for 24 h in PFA and thereafter dehydrated in increasing ethanol dilutions until 100%, followed by embedment in paraffin. Coronal section (8 μm) were cut at hippocampal level (comparable to bregma − 1.85 mm for adult mouse brain; Supplementary Fig. 1).

To assess lesion size, sections were deparaffinized and endogenous peroxidase was blocked with 3% H_2_O_2_ (VWR) in methanol for 20 min. Sections were rehydrated and antigen retrieval was performed in 10 mM citrate buffer (pH 6.0) for 3 min at 95 °C. Slides were cooled on ice for 30 min and washed with PBS. Aspecific binding of the antibody was blocked with 5% Normal Horse Serum (NHS, 26050088, Thermofisher Scientific) in PBS for 30 min at 37 °C. Primary antibody incubation with mouse anti-Microtubule-associated protein 2 (MAP2, 1:1000, M4403, Merck KgA) was performed overnight in 2% NHS-PBS solution. The next day, slides were washed with PBS. Secondary biotin-conjugated horse-anti-mouse antibody (1:100, BA2000, Vector Laboratories, Newark, USA) was diluted in PBS and incubated for 45 min at 37 °C. After incubation, sections were washed in PBS and incubated with vectastain ABC kit (PK-6100, Vector Laboratories) for 30 min at Room Temperature (RT) followed by incubation in 200 mL 3,3’-diaminobenzidine (DAB) solution with 60μL 30% H_2_O_2_ in 0.05 M Tris-HCl (pH 7.6, Roche, Basel, Switzerland) for 3 min. Lastly, slides were dehydrated in increasing ethanol dilutions and xylene, and embedded with DEPEX. Full-section images were captured with a Nikon D1 digital camera (Nikon, Tokyo, Japan). Area measurements of the MAP2^+^ area in ipsilateral and contralateral hemispheres were performed by a blinded observer using Adobe Photoshop CS6 and ipsilateral MAP2 loss was calculated as [1 - (ipsilateral MAP2^+^ area/ MAP2^+^ contralateral area) x 100%].

To assess glial scarring and mature neuron numbers, sections were deparaffinized, rehydrated and antigen retrieval was performed as described above. Non-specific binding was blocked by incubation in blocking buffer consisting of 2% Bovine Serum Albumin (BSA, A3059-100G, Merck KgA) and 0.1% Saponin (#S7900-25G, Merck KgA) in PBS. Next, sections were incubated overnight at 4 °C with primary antibody against mouse-anti-Glial fibrillary acidic protein (GFAP, 1:100, BM2287, Acris, Herford, Germany) and rabbit-anti-Hexaribonucleotide Binding Protein-3 (NeuN, 1:500, ab177487, Abcam, Cambridge, MA, USA) in PBS. The next day, sections were washed with PBS followed by incubation with Alexafluor 488-conjugated anti-mouse secondary antibody and Alexafluor 594-conjugated anti-rabbit secondary antibody (1:500, A11012, A11001, Thermofisher Scientific) for 1 h at RT and counterstained with DAPI (1:5000) for 5 min. After washing in H_2_O, sections were embedded in FluorSave (345789, VWR). Fluorescent images were taken using an Axio Observer Microscope at 10x magnification with Zen software equipped with AxioCam MRm. One image was analyzed for the dorsal (cortical region) measurement, and two images were averaged for the ventral (thalamic region) measurement (Fig. 5A). NeuN^+^ cell count and GFAP^+^ signal per mm^2^ were measured by a blinded observer using FIJI software v1.53.

### ELISA

At 10 or 17 days post-HI, HI and sham-control littermates were sacrificed by overdose pentobarbital followed by decapitation. Brains were quickly removed and contra- and ipsilateral hemispheres were separately snapfrozen in liquid nitrogen and stored at -80 °C until use. Ipsilateral hemispheres were crushed on liquid nitrogen and powdered brain tissue was homogenized in lysis buffer containing 10 mL D-PBS and 1 Complete tablet Mini EDTA free (11836170001, Roche) in gentleMACS™ M Tubes using the OctoDissocator (Miltenyi Biotec B.V., Leiden, The Netherlands). Afterwards, samples were centrifuged for 10 min at 3220 g at 4 °C. Next, supernatants were sonicated on ice 2 times for 5 s with 30 s rest in between cycles, followed by centrifugation for 10 min at 4 °C at 3220 g. Supernatant was collected and protein level was quantified with the Bradford protein assay (500-0006, Bio-Rad, Ede, The Netherlands) following the manufacturer’s instructions with BSA as a standard for the reference curve. Absorbance was measured at 595 nm, using SKanIt™ (Thermo Fisher Scientific). CXCL10 levels were measured with an IP-10 (CXCL10) Mouse ELISA Kit (BMS6018, Merck KgA) following the manufacturer’s instructions.

### BV2 cell culture

Mouse BV2 microglia (ATCC, Gaithersburg, USA) were cultured in medium containing DMEM with glucose and without pyruvate (11,995,081, Fisher Scientific), 10% FCS, 1% GlutaMax, and 1% P/S in T75 flasks and incubated at 37 °C, 5% CO_2_, and 90% humidity and passaged once before use. BV2 cells were used as control cells for CXCR3 PCR and Western Blotting, see below.

### qPCR

MSCs and BV2 microglia were cultured as described above. For RNA collection, two T75 flasks of cells were pooled per condition. Flasks were first washed with ice-cold D-PBS followed by addition of 3 mL of Trizol (15596018, Thermo Scientific) to each flask in which cells were collected on ice by using a cell scraper. RNA isolation was performed according to the manufacturer’s instructions. The amount of RNA per sample was measured with the Nanodrop 2000 (Thermo Fisher Scientific). cDNA was synthesised with the iScript reverse transcription supermix (1,708,841, Bio-Rad) according to the manufacturer’s instructions. The expression of *cxcr3* (see Table 1 for primer sequences) was measured by qPCR (Quantstudio 3, Thermo Fisher Scientific). cDNA samples were diluted 40 times in RNA-free H_2_O, and Sybr^™^ Select Master Mix (13266519, Thermo Fisher Scientific) was used. q-PCR products were electrophoresed on 2% agarose gel (11388991001, Merck KGaA), which were stained with 35 mg of Orange G (V000846, Merck KGaA) in 30% glycerol (G5516 Merck KGaA) in ddH_2_O (B230531, Fresenius Kabi AG, Bad Homburg, Germany) and imaged with an Amersham IQ800 Imager (Proxima 2750, Cytiva Life Sciences, Medimblik, The Netherlands).

**Table 1:** Primers used for qPCR (Merck KGaA)

Name	Sequence forward (5’ --> 3’)	Sequence reverse (3‘--> 5’)
*cxcr3*	TCAGCCAACTACGATCAGCG	CCTCTGGAGACCAGCAGAAC

### Western blotting

MSCs and BV2 microglia were cultured as described above. Three T75 flasks of cells were pooled to isolate cytosolic and plasma membrane protein fractions for western blot analysis. Flasks were washed with ice-cold dPBS and cells were harvested on ice with a cell scraper in cell fraction lysis buffer, containing 20mM Tris-HCl, 2mM EDTA (pH 8, 798681-1KG, Merck KGaA), 1 tablet Complete tablets Mini EDTA-free and the phosphatase inhibitors NaF (5mM) and Na_3_VO_4_ (1mM). Cell lysate was collected, sonicated for 5 s on ice, and spun down at 2200 RPM for 5 min at 4 °C. Supernatant was spun down further at 32.000 RPM in an ultracentrifuge (OPTIMA TLX 9702, CTX97H02, Beckman, Brea, USA) for 20 min at 4 °C. Supernatant was stored as the cytosol fraction at -20 °C. 50–100μL of pellet lysis buffer was added to the pellet depending on the pellet size, which contained 20mM Tris-HCl (pH 7.5), 2mM EDTA (pH 8), 1% TritonX100 (1.086.031.000, VWR), Mini EDTA free tablets, and the phosphatase inhibitors as described above. Pellets were resolved by sonicating three times for 5 s on ice and stored as plasma membrane fraction at -20 °C until further use. Protein levels were quantified with the Bradford protein assay as described above. 15 μg of protein was loaded for each sample onto a 4–20% Criterion™ TGX™ precast gel (12 + 2 well, 45μL, 5671093, Bio-Rad). Protein samples were separated by SDS-PAGE and transferred to nitrocellulose membrane (1620168, Bio-Rad). The membrane was washed for 5 min with tris-buffered saline (washing buffer) containing 0.1% Tween (VWR) in demi water. Next, the membrane was blocked for 1 h in washing buffer with 5% BSA. After blocking the membrane was incubated overnight with 1:1000 rabbit-anti-CXCR3 (ab71864, Abcam) or 1:10.000 mouse-anti-beta-actin (A5316, Merck KGaA) in washing buffer with 5% BSA. Next day, the blot was washed three times with washing buffer and incubated with 1:5000 secondary antibody HRP-linked-anti-rabbit IgG (NA934V, GE healthcare, Chicago, Illinois) or HRP-linked-anti-mouse IgG (NA931V, GE Healthcare) for 1 h at RT. Imaging was performed using ECL (RPN2106, Fisher Scientific) according to manufacturer’s instructions, on an Amersham IQ800 Imager (Cytiva Life Sciences).

### Transwell migration assay

Migration of MSCs towards CXCL10 was assessed in vitro via a transwell migration assay. 2.5 × 10^4^ MSCs were resuspended in 100 μL serum-free medium (31331093, Merck KGaA) and loaded in the upper part of transwell membrane inserts (8 μm pore size, CLS3422, Merck KGaA) in a 24-wells plate (Corning). In the well, 600 μL DMEM-F12 was added, which contained 0.1% FCS in all conditions. The negative control wells contained 0.1% Dimethyl sulfoxide (DMSO, 4720.4, Brunschwig, Amsterdam, The Netherlands) and 0.017% BSA, and the positive control wells contained 100 ng/mL CXCL10 in 0.017%BSA. MSCs were pre-exposed to CXCR3-inhibitors AMG487 (1μM, HY-15319, MedChemExpress, Solentuna, Sweden) or NBI-74,330 (100nM, HY-15320, MedChemExpress) diluted in 0.1% DMSO for 45 min before adding the MSCs to the transwell insert. Additionally, similar concentrations of the inhibitors were added to the wells. MSCs were allowed to migrate for 6 h in the incubator before fixation with ice-cold methanol (PROL20903.368, VWR) in a 24-wells plate. All non-migrated cells were removed with a cotton tip from the upper side of the membrane. Migrated MSCs at the bottom of the membrane were stained by adding 1:5000 DAPI in dH_2_O to the wells for 5 min at RT. Membranes were detached from the insert, and mounted cell side up on Superfrost Plus slides (6310108, VWR) with one drop of Fluorsave reagent and covered with high precision coverslips 24 × 50 mm #1,5 H (630–2187, VWR). Four pictures at distinct set locations were taken per membrane with 20x magnification on an Axio Observer Microscope equipped with AxioCam MRm and Zen software. DAPI-positive cells were counted in FIJI 1.53 by using the Particle Analyse plugin.

### Statistical analysis

Statistics were performed with GraphPad Prism 8.3 (Graphpad Software, Boston, MA, USA). Outliers were identified using the ROUT method (Q = 1%). Data was checked for normal (Gaussian) distribution with the Shapiro-Wilk normality test. Data was statistically analysed by unpaired t-test (in case of two groups) or one-way ANOVA (in case of three or more groups) and by Holm-Sidak post-hoc comparisons. Differences of *p* < 0.05 were deemed statistically significant. Experimental unit was either animal or cell culture well.

## Results

### Intranasal MSC therapy is not effective in repairing the lesion when administered at 17-days post-HI

In a previous study we showed that intranasal MSC treatment at day 3 and 10 after HI in neonatal mice was effective in reducing lesion size and improving motor impairment but that MSC treatment lost its efficacy when given at day 17 [10]. Here we confirm that intranasal MSC therapy given at 17 days post-HI (Fig. 1A) did not result in a beneficial effect on lesion size. HI animals showed around 30% grey matter damage in the ipsilateral hemisphere as assessed by loss of MAP2 staining (*p* < 0.0001 compared to sham-controls). MSC therapy at day 17 post-HI did not reduce MAP2 loss (*p* = 0.8736, Fig. 1B-C) indicating that this timing of MSC treatment is outside the therapeutic window.

### MSC migration into the brain after intranasal delivery is more efficient at day 10 than at day 17 post-HI

To assess why MSC therapy is not effective when started at 17 days post-HI [10], MSC migration into the brain was determined at day 10 or day 17 post-HI. Our previous study qualitatively indicated reduced migration of MSCs at later timepoints post-HI [10]. Here we quantified migration of PKH26-labeled MSCs after intranasal delivery at day 10 or day 17 post-HI by tracing the PKH26^+^ signal around the lesion site at 24 h after administration (Fig. 1D). A substantial amount of PKH26-signal was detected around the lesion in animals treated at 10 days post-HI (Fig. 1E-F). In animals treated at 17 days the PKH26^+^ signal was strongly reduced when compared to 10 days post-HI (*p* = 0.006, Fig. 1E-F). These data indicate that the loss of efficacy of MSC therapy at later timepoints post-HI might be caused by fewer MSCs reaching the brain after intranasal delivery.

### Cerebral CXCL10 protein levels are lower at day 17 compared to 10 days post-HI

In our previous work we showed, using a PCR array, that various chemotactic factors were differentially expressed in the HI brain lesion milieu at day 10 compared to day 17 post-insult [9]. Since MSC migration is dependent on chemotactic factors, the potential role of one of the identified chemokines, CXCL10, in the migration of MSCs was assessed (Fig. 1G). Compared to sham-control brains, a significant increase in CXCL10 protein levels was observed in the ipsilateral hemisphere of HI brains at 10 days post-insult (*p* < 0.0001). At day 17 post-HI, ipsilateral CXCL10 levels were significantly reduced compared to day 10 post-HI (*p* = 0.0008, Fig. 1H).

### CXCL10 induces MSC migration via the CXCR3 receptor

To investigate the effect of CXCL10 on MSC migration we performed an in vitro transwell migration assay. First, mRNA expression of the receptor for CXCL10, i.e. *cxcr3*, on murine BM-MSCs was confirmed by qPCR. Both the positive control sample of a BV2 cell line and MSCs showed qPCR products as expected around 170 basepairs (Fig. 2A). Additionally, we confirmed that MSCs express CXCR3 on the plasma membrane by using Western Blotting: positive bands at 40 kDa and 80 kDa were observed, indicating that MSCs indeed express CXCR3 under basal conditions (Fig. 2B). The same kDa bands were found in the positive control BV2 sample which shows CXCR3 expression at a high basal level compared to MSCs. Next, we performed an in vitro transwell experiment to study MSC migration towards CXCL10. Without CXCL10 in the lower compartment, MSCs showed limited migration across the membrane of the transwell insert (Fig. 2C). After dose-response optimization (data not shown), addition of the optimal concentration of 100 ng/mL CXCL10 to the lower compartment significantly increased MSC migration compared to the negative control (*p* < 0.0001, Fig. 2C). To confirm that MSC migration towards CXCL10 was mediated by CXCR3, MSCs were preincubated with CXCR3 inhibitors AMG487 or NBI-74330, resulting in significantly less MSC migration towards CXCL10 (*p* = 0.0430 AMG487, *p* = 0.0110 NBI-74330, Fig. 2C). In presence of the CXCR3 inhibitors, MSC migration was not significantly different from the negative control without CXCL10 (Fig. 2C). These data indicate that the CXCR3 receptor on MSCs regulates migration towards CXCL10.

### CXCL10 enhances MSC migration towards the HI-injured brain

To prove that CXCL10 is a major chemoattractant for MSCs towards the HI lesion after intranasal delivery, CXCL10 was intracranially injected in the lesion at 17 days post-HI (Fig. 3A), i.e., the timepoint at which MSC migration was strongly reduced (Fig. 1E/F). Within 6 h after CXCL10 injection, PKH26^+^ MSCs were administered intranasally and 24 h later brains were collected to quantify PKH26^+^ signal in the brain parenchyma around the lesion (Fig. 3A). Significantly more PKH26^+^ signal was detected around the HI lesion after intracranial CXCL10 injection compared to PBS injection (*p* = 0.0008, Fig. 3B-C). These results show the crucial role of elevated CXCL10 levels in the lesion for regulating MSC migration towards the HI-injured brain.

### CXCL10 restores therapeutic efficacy of late intranasal MSC treatment on anatomical but not functional level

Since increased CXCL10 levels in the lesion at day 17 post-HI reinforces MSC migration (Fig. 3B-C), we next assessed whether intracranial CXCL10 injection can also restore the treatment efficacy of delayed MSC therapy with the lowest effective dose [10]. To this end, intranasal MSCs or PBS were administered following intracranial CXCL10 injection at day 17 post-HI and the effect of MSC treatment on lesion size and motor impairment was assessed 18 days later (Fig. 4A). HI animals that received intracranial CXCL10 without MSC treatment (i.e., i.c. CXCL10 + i.n. PBS) showed 33% ipsilateral MAP2 loss (*p* < 0.0001 compared to SHAM-operated controls, Fig. 4B-C). Intranasal MSC therapy following intracranial CXCL10 injection significantly reduced ipsilateral grey matter damage to 20% (*p* = 0.0229 comparison to i.c. CXCL10 + i.n. PBS group, Fig. 4B-C). These data indicate that the CXCL10-enriched brain milieu can reinforce repair of the HI lesion by intranasal MSC therapy at late timepoints after HI (Fig. 1B-C). However, although ipsilateral grey matter damage was reduced, intranasal MSC treatment at 17 days post-HI did not reduce motor impairment in CXCL10-enriched HI animals (*P* = 0.6783 compared to i.c. CXCL10 + i.n. PBS animals; Fig. 4D).

### CXCL10 restores regenerative efficacy of late MSC treatment by reducing glial scarring and increasing neuronal numbers around the lesion

To further examine the anatomical improvements of the CXCL10-enriched lesion after intranasal MSC treatment at day 17 post-HI, we assessed glial scar formation and defined the number of neurons around the lesion [8] (Fig. 5A). GFAP^+^ signal and the number of NeuN^+^ neurons were quantified in two ventral thalamic regions and one dorsal cortical region around the lesion as indicated in Fig. 5A. GFAP^+^ glial scarring was present in brains of CXCL10-enriched HIE animals that received intranasal PBS treatment (thalamus: *p* < 0.0001 (C); cortex: *p* < 0.001 (D), Fig. 5B-D). Intranasal MSC treatment significantly reduced GFAP^+^ signal in both regions (thalamus: *p* = 0.0004 (C), cortex: *p* = 0.0068 (D) versus i.c. CXCL10 + i.n. PBS; Fig. 5B-D). Specifically in the thalamic region, less NeuN^+^ neurons were present around the lesion in brains of CXCL10-enriched HIE animals that received intranasal PBS treatment compared to SHAM animals (*p* = 0.0394, Fig. 5B, E). Intranasal MSC treatment significantly increased the number of NeuN^+^ neurons in the thalamic perilesional area (*p* = 0.0260 compared to i.c. CXCL10 + i.n. PBS, Fig. 5B, E). NeuN^+^ numbers were not affected by HI or treatment in the cortical region (*p* = 0.4776, Fig. 5B, F). These data together indicate that enhancing perilesional CXCL10 levels enables intranasally applied MSCs to reduce the glial scar and to increase neuron numbers in the perilesional (mostly thalamic) regions.

## Discussion

In this study we investigated the role of CXCL10 in the migration of MSCs towards the neonatal HI injured brain after intranasal delivery in mice. Our findings show that HI-induced ipsilateral CXCL10 levels in the brain were strongly reduced at day 17 compared to day 10 post-HI. These results are consistent with other studies on neonatal HI brain injury indicating an increase in CXCL10 following the HI insult and a subsequent decrease over time [21, 29]. CXCL10 is one player in a tightly regulated network of chemokines expressed after HI injury. Chemokines, such as C-X-C motif chemokine Ligand 12 (CXCL12), Chemokine C-C motif Ligand 4 (CCL4) or Chemokine C-C motif Ligand 5 (CCL5), exhibit similar patterns as CXCL10, being upregulated up to 10 days post-HI injury, suggesting a potential role for these chemokines in guiding MSCs towards the injury site after intranasal application [9, 30, 31]. In this study, we focused on CXCL10 since the expression of this chemokine was strongly reduced between day 10 and day 17 post-HI, corresponding with the time frame in which intranasal MSC treatment lost its efficacy [9]. The involvement of other chemokines in MSC migration could be the focus of future studies.

As outlined in this study, reduced cerebral CXCL10 levels coincided with a decrease in MSC migration to the HI lesion when MSCs were administered at 17 days post-HI. Notably, injecting CXCL10 perilesional before intranasal MSCs administration at day 17 post-HI, partially rescued MSC migration to the ipsilateral hemisphere, suggesting that CXCL10 plays a crucial role in guiding MSCs towards the HI lesion in vivo. However, strategies to enhance CXCL10 levels in the brain to widen the therapeutic window for intranasal MSC therapy do not seem ideal for clinical application, as elevated levels of perilesional CXCL10 might worsen brain injury by increasing immune cell influx and hyperexcitability, potentially tweaking MSCs’ mode of action and by reducing sprouting of neurons [22, 32–37]. Our findings do imply the potential option to use CXCL10 as a biomarker to predict intranasal MSC migratory efficacy in a personalized manner rather than relying on a general estimation of the therapeutic window of intranasal MSC therapy. Elevated CXCL10 levels have been found in the cerebrospinal fluid of patients with neonatal post-hemorrhagic hydrocephalus and in serum of neonatal and adult patients with perinatal asphyxia and intracerebral hemorrhage respectively [38–40]. However, it is unclear whether CXCL10 levels in serum or cerebrospinal fluid correlate one-to-one to CXCL10 levels in the brain of patients [40]. Therefore, further research should focus on the predictive value of serum or cerebrospinal fluid CXCL10 levels as possible biomarkers to determine the treatment efficacy of MSC therapy.

Our study is the first to demonstrate increased migration of intranasally applied MSCs towards CXCL10 in vivo in a neonatal HI mouse model, which aligns with previous in vitro data showing that human BM-MSCs can migrate towards CXCL10 [25, 26, 41]. In our in vitro study, we demonstrate that murine BM-MSCs express the CXCR3 receptor under basal conditions and that inhibition of the CXCR3 receptor inhibits migration towards CXCL10, showing the involvement of the CXCR3 receptor in CXCL10-guided migration. Moreover, earlier findings of Donega et al. show that human BM-MSCs can respond to the HI brain environment at 10 days post-HI by increasing CXCR3 receptor expression [9], indicating that when MSCs reach the brain they could be optimally guided towards the lesion by upregulating CXCR3 receptors. Therefore, enhancing the expression of the CXCR3 receptor on MSCs may be a strategy to optimize MSC migration towards the naturally expressed CXCL10 in the HI lesion. CXCL10 is an Interferon-Gamma (IFN-γ)-inducible protein, and the expression of CXCL10/CXCR3 in MSCs can be enhanced through exposure to IFN-γ [41, 42]. IFN-γ preconditioning of MSCs prior to application has shown promise in animal models of periventricular leukomalacia and experimental autoimmune encephalomyelitis [43, 44] suggesting it might be a good approach to enhance migratory capacity and efficacy of MSC therapy in situations when CXCL10 levels are declining in the brain. Importantly, CXCL9 and CXCL11 are also IFN-γ-inducible chemokines that interact with the CXCR3 receptor and can potentially influence CXCR3-guided MSC migration towards the HI lesion [42]. Therefore, more research is needed, not only to further examine the mechanisms behind CXCR3-mediated MSC migration and CXCL10 expression in the HI brain, but also to explore the potential roles of CXCL9 and CXCL11 on MSC migration. Furthermore, whether enhancing CXCR3 expression on MSCs from other sources, like adipose tissue or umbilical cord tissue, would have similar beneficial effects on migratory capacity would be an interesting topic for future research, as specific chemokine receptor expression levels might differ per MSC source [45–47].

In this study PKH26 labelling was used to trace MSCs in the HI-injured brain. While higher PKH26^+^ signal indicates a higher number of MSCs reaching the brain [48], a limitation of this method is that PKH26 signal does not distinguish between live MSCs, MSC-derived exosomes and MSC components engulfed by other cells [48, 49]. The indication of PKH26 + signal around the HI lesion indicates that at least part of the labeled MSCs or MSC-derived exosomes reached the brain after 24 h. Intranasal delivery of MSCs in neonatal brain injury models has been confirmed using other tracing techniques including MPIO-labeling with MR imaging and gold nanoparticle labeling with mass spectrometry [8, 50]. Nevertheless, in our study, not only PKH26^+^ signal is increased after i.c. CXCL10 injection at 17 days post-HI, but MSCs are still capable of inducing repair of the HI lesion when administered in a CXCL10-enriched milieu. Our data therefore indicate that live MSCs or MSC-derived exosomes have exerted their effects, at least for a period long enough to boost repair.

Our study shows that MSCs can activate neurorepair even at a relative late timepoint of 17 days post-HI. This is in alignment with other studies using models for neonatal HI that demonstrate activation of neurogenic niches (assessed as an enlargement of the subventricular zone and increased cell proliferation) up to postnatal day 31 [51, 52]. In addition, proliferating immature doublecortin^+^ cells have been observed around the lesion site until 2 months after HI injury [52, 53]. However, at late timepoints (P31/ 5-weeks) post-HI, almost no new adult neurons survived, indicating that newly born neurons may die soon due to a lack of trophic support in the HI micro-environment and because of hindrance of repair by the established glial scar that impedes new axonal outgrowth and network repair [52, 53]. MSCs, especially at later timepoints, might provide this trophic support and reduce glial scarring [8]. In this study, we found increased numbers of NeuN^+^ cells and a reduction of the GFAP^+^ glial scar around the lesion after i.c. CXCL10 injection followed by MSCs administration at day 17 post-HI. Other studies have shown that MSC or MSC-extracellular vesicle administration effectively reduces astrocytic activation when administered after the acute phase, potentially via the secretome or cargo [8, 54]. Since the glial scar is already formed prior to day 17 after HI [8], we show here that the glial scar can still be actively reversed by delayed MSC treatment possibly by the MSCs’ secretome tweaking the HI micro-environment. Specifically, MSC-derived Interleukin-6 (IL-6) has been shown to reduce reactive astrocyte proliferation after HI injury in rats [55]. It is likely that MSCs secrete a plethora of modulatory factors, including IL-6, which actively contribute to the resolution of existing glial scarring. Nevertheless, there may be limits to further extending the therapeutic window for intranasal MSC therapy, attributable to a potential point of no-return in glial scar formation, the inherent decline in brain plasticity and the reduction in the brain’s endogenous regenerative capacity [56–58]. More research is needed to determine the limits to which the therapeutic window of MSC therapy can be extended after neonatal HI injury.

In contrast to the beneficial effect of delayed MSC treatment on lesion size, we did not find significant functional recovery after MSC administration combined with intracranial CXCL10 at day 17 post-HI. MSC treatment at 10 days post-HI has been shown to enhance the connectivity between the impaired forepaw and the ipsilesional motor cortex after HI, which is related to improved sensorimotor function [59]. Delayed administration of MSCs might be too late to alter this connectivity in the corticospinal tract. Moreover, CXCL10 injection late after injury might lead to a flare up of inflammation in the lesion microenvironment and possible influx of immune cells [22, 32–37], to which arriving MSCs likely respond by adapting their secretome [50, 60]. Consequently, MSCs in the CXCL10-enriched micro-environment might exert an increased immunomodulatory function that can indirectly support repair, but which might not perfectly match the temporal regenerative and trophic need of the damaged brain areas and tracts to significantly restore functional outcome after late MSC treatment. Additionally, CXCL10 enrichment does not completely rescue MSC migration towards the HI lesion, which could also hamper the effect on functional repair. Therefore, a higher dosage of MSCs might be required at delayed treatment timepoints to improve functional repair. Interestingly, we did observe a high variability in the effect on motor performance after i.c. CXCL10 + i.n. MSC treatment indicating that delayed MSC therapy might induce sensorimotor improvement in at least part of the animals. Therefore, comprehensive testing in other functional domains could be interesting to elucidate if the combination of CXCL10 and late MSC treatment could induce functional repair in specific behavioral domains.

In conclusion, our study demonstrates that CXCL10 is a crucial chemokine for MSC migration towards the HI brain. Increased levels of CXCL10 drive effective MSC delivery and repair of the HI lesion beyond the therapeutic window of 10 days post-HI. Harnessing CXCR3-guided migration of MSCs offers possibilities to enhance the efficacy of MSC therapy to treat neonates with HI brain injury in the future.

### Electronic supplementary material

Below is the link to the electronic supplementary material.

**Supplementary Material 1: Supplementary Fig. 1**: location of stereotactic injection with CXCL10 or PBS in the ipsilateral hemisphere of HI-injured mice. Adapted from Mouse Brain Atlas (gaidi.ca) red dot shows location of injection.

**Supplementary Material 2: Supplementary Fig. 2**: full-length gel and blot of CXCR3 receptor expression. A)qPCR product of CXCR3 transcript in BV2 cells (positive ctrl) and MSCs. Arrow indicates representative qPCR product of 170 bp. Red square indicates cropped gel in Fig. 2.B)Western blot showing CXCR3 protein expression in the plasma membrane of MSCs. Protein bands corresponding to CXCR3 at 40 kDa and 80 kDa in BV2 cells (positive ctrl) and MSCs, indicated by the arrows. Red square indicated cropped blot in Figure. 2.

## Data Availability

The datasets used and/or analyzed during the current study are available from the corresponding author on reasonable request.

## Abbreviations

BM: Bone marrow
bp: Base pair
BSA: Bovine Serum Albumin
CCL4: Chemokine C-C motif ligands 4
CCL5: Chemokine C-C motif Ligands 5
CRT: Cylinder Rearing Task
CXCL10: Chemokine-X-C motif chemokine Ligand 10
CXCL11: C-X-C motif chemokine Ligand 11
CXCL12: C-X-C motif chemokine Ligand 12
CXCL9: C-X-C motif chemokine Ligand 9
CXCR3: C-X-C Motif Chemokine Receptor 3
DAPI: 4′6-diamidino-2-phenylindole
DMSO: Dimethyl Sulfoxide
Erk1/2: Extracellular signal-regulated protein kinase 1 and 2
FCS: Fetal Calf Serum
GFAP: Glial Fibrillary Acidic Protein
HI: Hypoxia-Ischemia
HIE: Hypoxic-Ischemic Encephalopathy
i.c.: Intracranial
i.n.: Intranasal
IFN-γ: Interferon-Gamma
IL-6: Interleukin-6
kDa: Kilodalton
MAP2: Microtubule-Associated Protein 2
MAPK: Mitogen activated protein kinase
MSC: Mesenchymal stem cell
NeuN: Hexaribonucleotide Binding Protein-3
NHS: Normal Horse Serum
P: Postnatal day
PBS: Phosphate-Buffered-Saline
PI3K: Phosphoinositide 3-kinase
RT: Room Temperature
Src: Proto-oncogene tyrosine-protein kinase Src

## References

[1] Greco P, Nencini G, Piva I, Scioscia M, Volta CA, Spadaro S (2020). Pathophysiology of hypoxic–ischemic encephalopathy: a review of the past and a view on the future. Acta Neurol Belg.

[2] Kurinczuk JJ, White-Koning M, Badawi N (2010). Epidemiology of neonatal encephalopathy and hypoxic-ischaemic encephalopathy. Early Hum Dev.

[3] Koper OM, Kaminska J, Sawicki K, Kemona H. CXCL9, CXCL10, CXCL11, and their receptor (CXCR3) in neuroinflammation and neurodegeneration. Advances in clinical and experimental medicine. Wroclaw University of Medicine; 2018. pp. 849–56. 10.17219/acem/68846 29893515

[4] Kariholu U, Montaldo P, Markati T, Lally P, Pryce R, Teiserskas J et al. Therapeutic hypothermia for mild neonatal encephalopathy: a systematic review and meta-analysis. Arch Dis Child Fetal Neonatal Ed. 2020;105. 10.1136/archdischild-2018-315711 30567775

[5] Tagin MA, Woolcott CG, Vincer MJ, Whyte RK, Stinson DA (2012). Hypothermia for neonatal hypoxic ischemic encephalopathy: an updated systematic review and Meta-analysis. Arch Pediatr Adolesc Med.

[6] Serrenho I, Rosado M, Dinis A, Cardoso CM, Grãos M, Manadas B (2021). Stem cell therapy for neonatal hypoxic-ischemic encephalopathy: a systematic review of preclinical studies. Int J Mol Sci.

[7] Musiał-Wysocka A, Kot M, Majka M. The pros and cons of mesenchymal stem cell-based therapies. Cell Transpl. 2019:801–12. 10.1177/0963689719837897 PMC6719501 31018669

[8] Donega V, Nijboer CH, van Tilborg G, Dijkhuizen RM, Kavelaars A, Heijnen CJ (2014). Intranasally administered mesenchymal stem cells promote a regenerative niche for repair of neonatal ischemic brain injury. Exp Neurol.

[9] Donega V, Nijboer CH, Braccioli L, Slaper-Cortenbach I, Kavelaars A, Van Bel F et al. Intranasal administration of human MSC for ischemic brain injury in the mouse: in vitro and in vivo neuroregenerative functions. PLoS ONE. 2014;9. 10.1371/journal.pone.0112339 PMC4232359 25396420

[10] Donega V, van Velthoven CTJ, Nijboer CH, van Bel F, Kas MJH, Kavelaars A (2013). Intranasal mesenchymal stem cell treatment for neonatal brain damage: long-term cognitive and Sensorimotor Improvement. PLoS ONE.

[11] Donega V, Nijboer CH, van Velthoven CT, Youssef Sa, de Bruin A, van Bel F (2015). Assessment of long-term safety and efficacy of intranasal mesenchymal stem cell treatment for neonatal brain injury in the mouse. Pediatr Res.

[12] Galeano C, Qiu Z, Mishra A, Farnsworth SL, Hemmi JJ, Moreira A (2018). The Route by which Intranasally delivered stem cells enter the Central Nervous System. Cell Transpl.

[13] Wei N, Yu SP, Gu X, Taylor TM, Song D, Liu XF (2013). Delayed intranasal delivery of hypoxic-preconditioned bone marrow mesenchymal stem cells enhanced cell homing and therapeutic benefits after ischemic stroke in mice. Cell Transpl.

[14] Herz J, Köster C, Reinboth BS, Dzietko M, Hansen W, Sabir H (2018). Interaction between hypothermia and delayed mesenchymal stem cell therapy in neonatal hypoxic-ischemic brain injury. Brain Behav Immun.

[15] van Velthoven CTJ, Kavalaars A, van Bel F, Heijnen CJ (2010). Nasal administration of stem cells: a promising Novel Route to. Pediatr Res.

[16] Vannucci SJ, Back SA (2022). The Vannucci Model of Hypoxic-Ischemic Injury in the neonatal rodent: 40 years later. Dev Neurosci.

[17] Wagenaar N, Nijboer CH, van Bel F (2017). Repair of neonatal brain injury: bringing stem cell-based therapy into clinical practice. Dev Med Child Neurol.

[18] Rappert A, Bechmann I, Pivneva T, Mahlo J, Biber K, Nolte C (2004). CXCR3-dependent microglial recruitment is essential for dendrite loss after brain lesion. J Neurosci.

[19] Koper OM, Kaminska J, Sawicki K, Kemona H (2018). CXCL9, CXCL10, CXCL11, and their receptor (CXCR3) in neuroinflammation and neurodegeneration. Adv Clin Experimental Med.

[20] Teo EJ, Chand KK, Miller SM, Wixey JA, Colditz PB, Bjorkman ST. Early evolution of glial morphology and inflammatory cytokines following hypoxic-ischemic injury in the newborn piglet brain. Sci Rep. 2023;13. 10.1038/s41598-022-27034-9 PMC9823001 36609414

[21] Jaworska J, Ziemka-Nalecz M, Sypecka J, Zalewska T. The potential neuroprotective role of a histone deacetylase inhibitor, sodium butyrate, after neonatal hypoxia-ischemia. J Neuroinflammation. 2017;14. 10.1186/s12974-017-0807-8 PMC5303300 28187734

[22] Muzio L, Cavasinni F, Marinaro C, Bergamaschi A, Bergami A, Porcheri C (2010). Cxcl10 enhances blood cells migration in the sub-ventricular zone of mice affected by experimental autoimmune encephalomyelitis. Mol Cell Neurosci.

[23] Cai W, Shi L, Zhao J, Xu F, Dufort C, Ye Q et al. Neuroprotection against ischemic stroke requires a specific class of early responder T cells in mice. J Clin Invest. 2022;132. 10.1172/JCI157678 PMC9337834 35912857

[24] Grygorczuk S, Osada J, Toczyłowski K, Sulik A, Czupryna P, Moniuszko-Malinowska A et al. The lymphocyte populations and their migration into the central nervous system in tick-borne encephalitis. Ticks Tick Borne Dis. 2020;11. 10.1016/j.ttbdis.2020.101467 32723646

[25] Kalwitz G, Andreas K, Endres M, Neumann K, Notter M, Ringe J (2010). Chemokine profile of human serum from whole blood: migratory effects of CXCL-10 and CXCL-11 on human mesenchymal stem cells. Connect Tissue Res.

[26] Rice CM, Scolding NJ (2010). Adult human mesenchymal cells proliferate and migrate in response to chemokines expressed in demyelination. Cell Adh Migr.

[27] Percie N, Hurst V, Id AA, Id SA, Id TA, Baker M (2020). The ARRIVE guidelines 2.0: updated guidelines for reporting animal research. PLoS Biol.

[28] Schallert T, Fleming SM, Leasure JL, Tillerson JL, Bland ST (2000). CNS plasticity and assessment of forelimb sensorimotor outcome in unilateral rat models of stroke, cortical ablation, parkinsonism and spinal cord injury. Neuropharmacology.

[29] Akamatsu T, Dai H, Mizuguchi M, Goto YI, Oka A, Itoh M (2014). LOX-1 is a novel therapeutic target in neonatal hypoxic-ischemic encephalopathy. Am J Pathol.

[30] Miller JT, Bartley JH, Wimborne HJC, Walker AL, Hess DC, Hill WD et al. The neuroblast and angioblast chemotaxic factor SDF-1 (CXCL12) expression is briefly up regulated by reactive astrocytes in brain following neonatal hypoxic-ischemic injury. BMC Neurosci. 2005;6. 10.1186/1471-2202-6-63 PMC1298306 16259636

[31] Hill WD, Hess DC, Martin-Studdard A, Carothers JJ, Zheng J, Hale D et al. SDF-1 (CXCL12) is upregulated in the ischemic Penumbra following stroke: association with bone marrow cell homing to injury. J Neuropathol Exp Neurol. 2004;63. 10.1093/jnen/63.1.84 14748564

[32] Chaitanya GV, Schwaninger M, Alexander JS, Babu PP (2010). Granzyme-b is involved in mediating post-ischemic neuronal death during focal cerebral ischemia in rat model. Neuroscience.

[33] Van Weering HRJ, Boddeke HWGM, Vinet J, Brouwer N, De Haas AH, Van Rooijen N (2011). CXCL10/CXCR3 signaling in glia cells differentially affects NMDA-induced cell death in CA and DG neurons of the mouse hippocampus. Hippocampus.

[34] Glaser J, Gonzalez R, Sadr E, Keirstead HS (2006). Neutralization of the chemokine CXCL10 reduces apoptosis and increases axon sprouting after spinal cord injury. J Neurosci Res.

[35] Petrisko TJ, Bloemer J, Pinky PD, Srinivas S, Heslin RT, Du Y et al. Neuronal CXCL10/CXCR3 axis mediates the induction of cerebral hyperexcitability by peripheral viral challenge. Front Neurosci. 2020;14. 10.3389/fnins.2020.00220 PMC7105801 32265633

[36] Sui Y, Stehno-Bittel L, Li S, Loganathan R, Dhillon NK, Pinson D (2006). CXCL10-induced cell death in neurons: role of calcium dysregulation. Eur J Neurosci.

[37] Cho J, Nelson TE, Bajova H, Gruol DL (2009). Chronic CXCL10 alters neuronal properties in rat hippocampal culture. J Neuroimmunol.

[38] Landreneau MJ, Mullen MT, Messé SR, Cucchiara B, Sheth KN, McCullough LD (2018). CCL2 and CXCL10 are associated with poor outcome after intracerebral hemorrhage. Ann Clin Transl Neurol.

[39] Habiyaremye G, Morales DM, Morgan CD, McAllister JP, CreveCoeur TS, Han RH (2017). Chemokine and cytokine levels in the lumbar cerebrospinal fluid of preterm infants with post-hemorrhagic hydrocephalus. Fluids Barriers CNS.

[40] Fotopoulos S, Mouchtouri A, Xanthou G, Lipsou N, Petrakou E, Xanthou M (2005). Inflammatory chemokine expression in the peripheral blood of neonates with perinatal asphyxia and perinatal or nosocomial infections. Acta Paediatr Int J Paediatrics.

[41] Juliana Croitoru-Lamoury, Francois MJ, Lamoury JJ, Zaunders LA, Veas BJ (2007). Brew. Human mesenchymal stem cells Constitutively Express chemokines and Chemokine receptors that can be upregulated by cytokines, IFN-B, and Copaxone. J Interferon Cytokine Res.

[42] Metzemaekers M, Vanheule V, Janssens R, Struyf S, Proost P. Overview of the mechanisms that may contribute to the non-redundant activities of interferon-inducible CXC chemokine receptor 3 ligands. Front Immunol. 2018;8. 10.3389/fimmu.2017.01970 PMC5775283 29379506

[43] Ahmadifard R, Jafarzadeh A, Mahmoodi M, Nemati M, Rahmani M, Khorramdelazad H (2022). Interferon-γ-Treated mesenchymal stem cells modulate the T cell-related chemokines and Chemokine receptors in an animal model of experimental autoimmune encephalomyelitis. Drug Res.

[44] Morioka C, Komaki M, Taki A, Honda I, Yokoyama N, Iwasaki K et al. Neuroprotective effects of human umbilical cord-derived mesenchymal stem cells on periventricular leukomalacia-like brain injury in neonatal rats. Inflamm Regen. 2017;37. 10.1186/s41232-016-0032-3 PMC5725779 29259700

[45] Heirani-Tabasi A, Toosi S, Mirahmadi M, Mishan MA, Bidkhori HR, Bahrami AR (2017). Chemokine receptors expression in MSCs: comparative analysis in different sources and passages. Tissue Eng Regen Med.

[46] Kia NA, Bahrami AR, Ebrahimi M, Matin MM, Neshati Z, Almohaddesin MR (2011). Comparative analysis of chemokine receptor’s expression in mesenchymal stem cells derived from human bone marrow and adipose tissue. J Mol Neurosci.

[47] Balasubramanian S, Venugopal P, Sundarraj S, Zakaria Z, Majumdar A, Sen, Ta M (2012). Comparison of chemokine and receptor gene expression between Wharton’s jelly and bone marrow-derived mesenchymal stromal cells. Cytotherapy.

[48] Nicholls FJ, Liu JR, Modo M (2017). A comparison of exogenous labels for the histological identification of transplanted neural stem cells. Cell Transpl.

[49] Li P, Zhang R, Sun H, Chen L, Liu F, Yao C (2013). PKH26 can transfer to host cells in vitro and vivo. Stem Cells Dev.

[50] Vaes JEG, van Kammen CM, Trayford C, van der Toorn A, Ruhwedel T, Benders MJNL (2021). Intranasal mesenchymal stem cell therapy to boost myelination after encephalopathy of prematurity. Glia.

[51] Felling RJ, Snyder MJ, Romanko MJ, Rothstein RP, Ziegler AN, Yang Z (2006). Neural stem/progenitor cells participate in the regenerative response to perinatal hypoxia/ischemia. J Neurosci.

[52] Plane JM, Liu R, Wang TW, Silverstein FS, Parent JM (2004). Neonatal hypoxic-ischemic injury increases forebrain subventricular zone neurogenesis in the mouse. Neurobiol Dis.

[53] Yang Z, Covey MV, Bitel CL, Ni L, Jonakait GM, Levison SW (2007). Sustained neocortical neurogenesis after neonatal hypoxic/ischemic injury. Ann Neurol.

[54] Kaminski N, Köster C, Mouloud Y, Börger V, Felderhoff-Müser U, Bendix I et al. Mesenchymal stromal cell-derived extracellular vesicles reduce neuroinflammation, promote neural cell proliferation and improve oligodendrocyte maturation in neonatal hypoxic-ischemic brain injury. Front Cell Neurosci. 2020;14. 10.3389/fncel.2020.601176 PMC7758466 33362471

[55] He M, Shi X, Yang M, Yang T, Li T, Chen J (2019). Mesenchymal stem cells-derived IL-6 activates AMPK/mTOR signaling to inhibit the proliferation of reactive astrocytes induced by hypoxic-ischemic brain damage. Exp Neurol.

[56] Hensch TK, Bilimoria PM. Re-opening windows: manipulating critical periods for brain development. Cerebrum. 2012:1–18. PMC3574806 23447797

[57] Ruddy RM, Morshead CM. Home sweet home: the neural stem cell niche throughout development and after injury. Cell Tissue Res. Springer Verlag; 2018. pp. 125–41. 10.1007/s00441-017-2658-0 28776186

[58] Wang Y, Chen X, Cao W, Shi Y (2014). Plasticity of mesenchymal stem cells in immunomodulation: pathological and therapeutic implications. Nat Immunol.

[59] van Velthoven C, van de Looij Y, Kavelaars A, Zijlstra J, van Bel F, Huppi P (2012). Mesenchymal stem cells restore cortical rewiring after neonatal ischemia in mice. Ann Neurol.

[60] Van Velthoven CTJ, Kavelaars A, Van Bel F, Heijnen CJ (2010). Repeated mesenchymal stem cell treatment after neonatal hypoxia-ischemia has distinct effects on formation and maturation of new neurons and oligodendrocytes leading to restoration of damage, corticospinal motor tract activity, and sensorimotor function. J Neurosci.

