# Supplementary figures and images for: CXCL10 is a crucial chemoattractant for efficient intranasal delivery of mesenchymal stem cells to the neonatal hypoxic-ischemic brain
Source: Stem Cell Res Ther. 2024 May 7;15:134. doi: 10.1186/s13287-024-03747-8 (PMC11077865; doi:10.1186/s13287-024-03747-8)

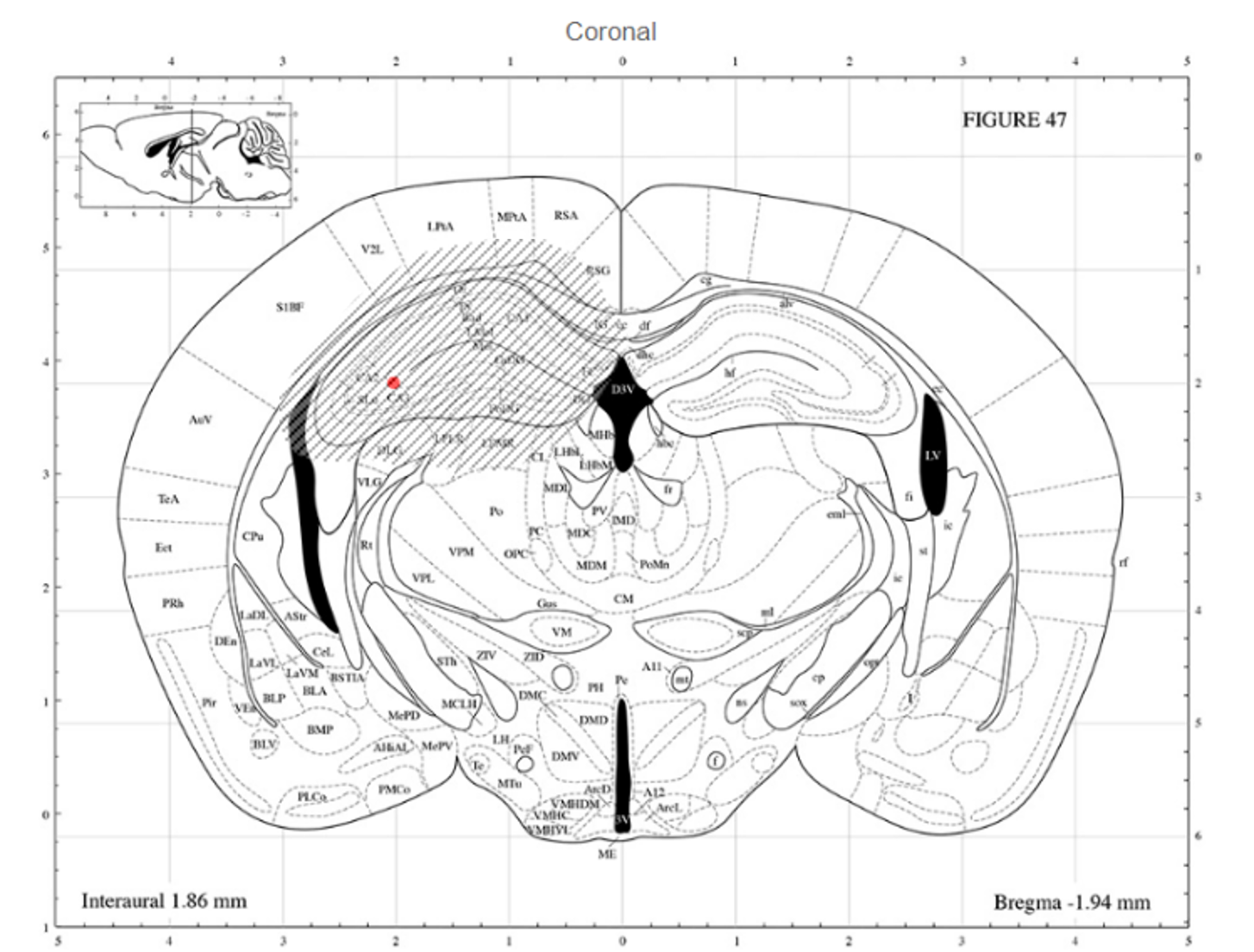

Supplement: Supplementary file 1 — Supplementary Material 1: Supplementary Fig. 1: location of stereotactic injection with CXCL10 or PBS in the ipsilateral hemisphere of HI-injured mice. Adapted from Mouse Brain Atlas (gaidi.ca) red dot shows location of injection. [file 13287_2024_3747_MOESM1_ESM.png]

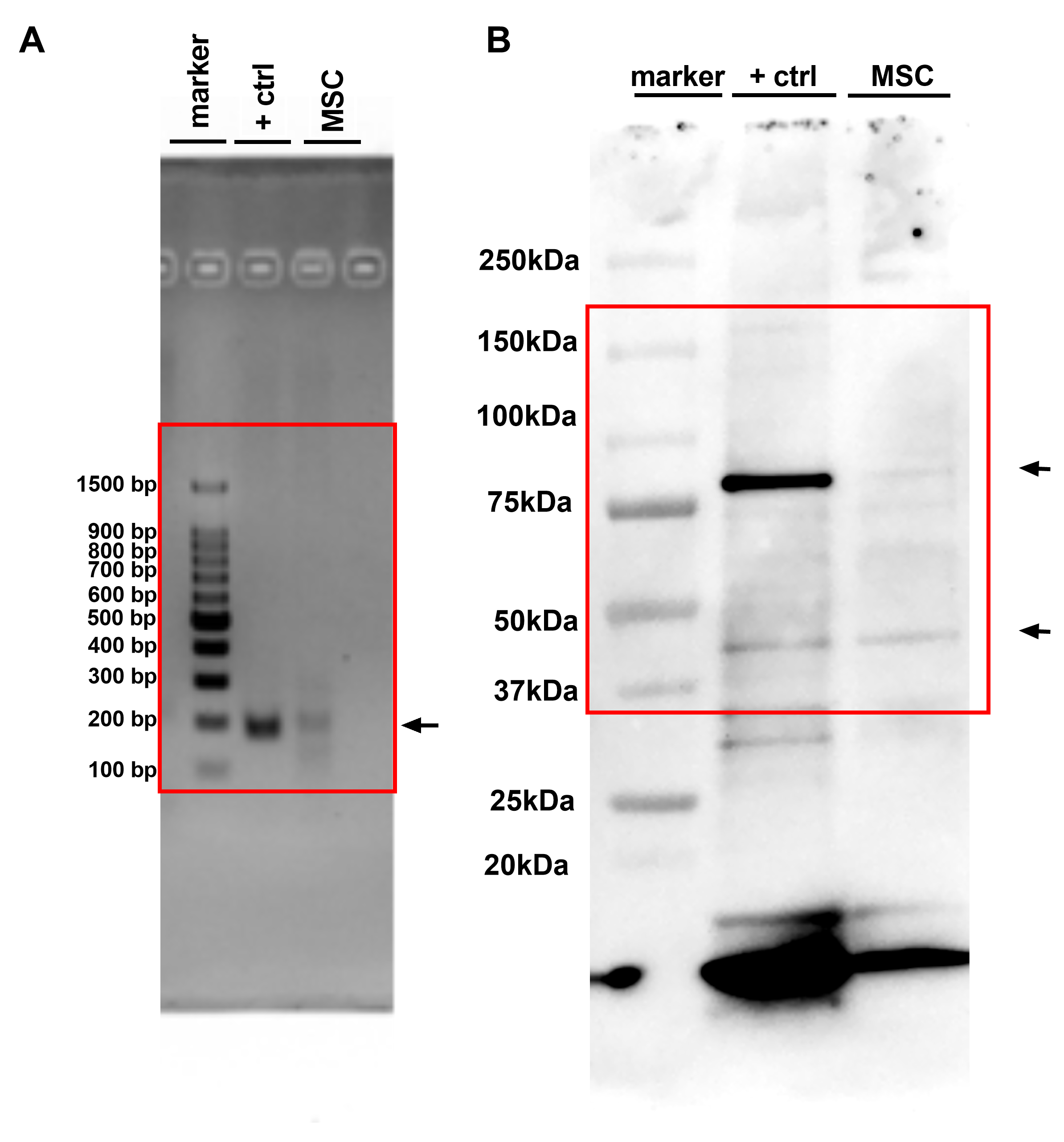

Supplement: Supplementary file 2 — Supplementary Material 2: Supplementary Fig. 2: full-length gel and blot of CXCR3 receptor expression. A)qPCR product of CXCR3 transcript in BV2 cells (positive ctrl) and MSCs. Arrow indicates representative qPCR product of 170 bp. Red square indicates cropped gel in Fig. 2.B)Western blot showing CXCR3 protein expression in the plasma membrane of MSCs. Protein bands corresponding to CXCR3 at 40 kDa and 80 kDa in BV2 cells (positive ctrl) and MSCs, indicated by the arrows. Red square indicated cropped blot in Figure. 2. [file 13287_2024_3747_MOESM2_ESM.png]
